# Gradient Waveguide Thickness Guided-Mode Resonance Biosensor

**DOI:** 10.3390/s21020376

**Published:** 2021-01-07

**Authors:** Jia-Ming Yang, Nien-Zu Yang, Cheng-Hao Chen, Cheng-Sheng Huang

**Affiliations:** Department of Mechanical Engineering, National Chiao Tung University, Hsinchu 30010, Taiwan; davidyang.me07g@nctu.edu.tw (J.-M.Y.); a0751025.me07g@nctu.edu.tw (N.-Z.Y.); ohyes12321.me07g@nctu.edu.tw (C.-H.C.)

**Keywords:** label-free biosensor, subwavelength grating, guided-mode resonance

## Abstract

Portable systems for detecting biomolecules have attracted considerable attention, owing to the demand for point-of-care testing applications. This has led to the development of lab-on-a-chip (LOC) devices. However, most LOCs are developed with a focus on automation and preprocessing of samples; fluorescence measurement, which requires additional off-chip detection instruments, remains the main detection method in conventional assays. By incorporating optical biosensors into LOCs, the biosensing system can be simplified and miniaturized. However, many optical sensors require an additional coupling device, such as a grating or prism, which complicates the optical path design of the system. In this study, we propose a new type of biosensor based on gradient waveguide thickness guided-mode resonance (GWT-GMR), which allows for the conversion of spectral information into spatial information such that the output signal can be recorded on a charge-coupled device for further analysis without any additional dispersive elements. A two-channel microfluidic chip with embedded GWT-GMRs was developed to detect two model assays in a buffer solution: albumin and creatinine. The results indicated that the limit of detection for albumin was 2.92 μg/mL for the concentration range of 0.8–500 μg/mL investigated in this study, and that for creatinine it was 12.05 μg/mL for the concentration range of 1–10,000 μg/mL. These results indicated that the proposed GWT-GMR sensor is suitable for use in clinical applications. Owing to its simple readout and optical path design, the GWT-GMR is considered ideal for integration with smartphones or as miniaturized displays in handheld devices, which could prove beneficial for future point-of-care applications.

## 1. Introduction

Label-free (LF) biosensors that can directly measure the concentration of target analytes without the need for fluorescence labels have numerous uses, such as in the diagnosis of diseases, monitoring of the environment, development of drugs, and the detection of biological warfare agents and chemicals [1,2]. Many different LF biosensors have been successfully manufactured; these biosensors have different transduction mechanisms, including optical, electrochemical, mechanical, and acoustic mechanisms [3]. LF biosensors based on optical resonance are among the most popular types of biosensors for quantifying biomolecules because of their high sensitivity, real-time monitoring and multiplexing capabilities, and simple fabrication and configuration [2,3,4]. Several optical devices have been employed as LF biosensors, including photonic crystals [5,6] and ring resonators [7]; LF biosensors that make use of phenomena such as surface plasmon resonance (SPR) [8], localized surface plasma resonance [9], and guided-mode resonance (GMR) [10,11] have also been developed. Among these phenomena, SPR has been the most widely used in LF biosensors. Many companies have developed biosensors based on SPR [12]. Alternatively, GMR-based biosensors, which have a high resolution and simple readout design, have also been widely investigated by researchers and commercialized by several companies as components of desktop systems for high-throughput applications [12]. Depending on the design of the light source and readout systems, researchers can measure the change in wavelength, intensity, coupling angle, or phase, and determine the association of such changes with changes in sample concentrations. Wavelength interrogation using a broadband light source and high-resolution spectrometers [11,13] is probably the most straightforward detection method. Detection techniques using tunable light sources and photodetectors [14,15,16], angle [10] and intensity [17] modulation, and phase interrogation [18] have also been successfully demonstrated.

Triggs et al. have designed a chirped GMR biosensor based on a graded duty cycle and used it to measure the binding between IgG and anti-IgG [19]. Chirped GMR allows for the conversion of spectral information into spatial information; hence, a charge-coupled device (CCD) or complementary metal–oxide–semiconductor (CMOS) sensor can be used to read out the signal output, which would eliminate the need for a bulky spectrometer. Such characteristics facilitate the integration of gradient GMR sensors with smartphones, without the need for any additional dispersive elements, such as a grating or prism.

Instead of using a graded duty cycle GMR sensor, previously, we used a gradient waveguide thickness GMR (GWT-GMR) sensor as a refractive index (RI) sensor [20]. GWT-GMR sensors can be fabricated inexpensively using replica molding on a plastic substrate. The subsequent deposition of the thickness gradient can be adjusted during the deposition process to achieve different sensor sensitivity, detection range, and resolution for different applications. In this study, we further optimized the incident wavelength through simulation software by determining the optimal wavelength based on a combination of sensitivity and resonant linewidth; the results were verified experimentally. Additionally, a two-channel microfluidic chip with embedded GWT-GMR sensors was fabricated. The measurement channel was used to measure the binding between immobilized antibodies and the target analytes. The reference channel was used to correct for any disturbance due to the measurement apparatus or surrounding environment. Finally, albumin and creatinine in buffer solutions were successfully quantified in a clinically relevant concentration range to demonstrate the feasibility of the biosensor.

## 2. Materials and Methods

**Figure 1:**
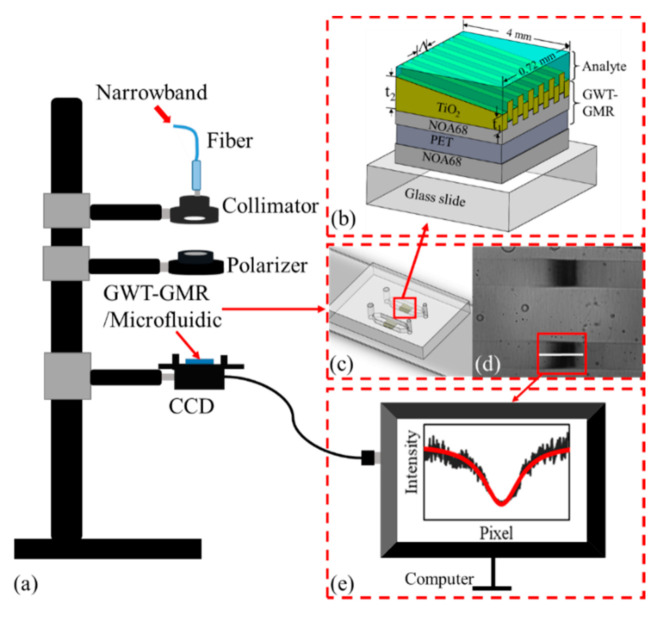
(**a**) Illustration of the transmission setup, (**b**) schematic of a gradient waveguide thickness guided-mode resonance (GWT-GMR) sensor bonded on a glass slide, (**c**) and two-channel microfluidic chip embedded with GWT-GMR sensors. (**d**) Examples of the transmission intensity distributions on a charge-coupled device (CCD). (**e**) The raw (black) and fitted (red) intensity distributions along a specific row of pixels, that is, the white line in (**d**).

Figure 1 shows the measurement system. The figure shows the optical setup for transmission measurement, the GWT-GMR sensor design and its integration with the two-channel microfluidic chip, and the setup for CCD imaging and signal analysis.

### 2.1. Design and Fabrication of the GWT-GMR Sensor

The main component of the GWT-GMR sensor is the GMR filter, which has been studied extensively since the late 1980s [21,22,23]. By appropriately designing the device geometry, such as the grating period, depth and duty cycle, and waveguide thickness as well as by selecting the appropriate materials, one can construct a GMR sensor that reflects a specific wavelength and transmits the rest of the wavelength at normal incidence [22,23,24]. Essentially, the GMR sensor functions as a bandstop filter whose center wavelength (or resonant wavelength, λ) is calculated using the second-order Bragg condition [25]:

(1) $$\lambda =n_{eff}\Lambda$$

where *n_eff_* is the effective RI of the structure, and Λ is the grating period. *n_eff_* can be considered as the weighted RIs, which are related to the RIs of the substrate, waveguiding layer, and cover (sample in this work) layer, and the thickness of the waveguiding layer.

Different designs and fabrication techniques have been adopted to achieve a gradient waveguide thickness such that a linear variable bandstop filter [20,26,27] is realized. The GWT-GMR sensor used in this study consists of three layers, as shown in Figure 1b; the layers include a substrate of polyethylene terephthalate (PET), a replicated grating structure of an ultraviolet (UV) adhesive (Norland 68, NOA68), and a TiO_2_ layer with gradient thickness. The detailed fabrication process can be found in a previous study [20]. In brief, the fabrication of the sensor mainly consists of three processes: electron-beam lithography (EBL), replica molding, and film deposition. We first used EBL and reactive-ion etching to form a 360 nm grating pattern with a grating depth of 85 nm and a duty cycle of 0.5 on a Si wafer. Then, the pattern was transferred to NOA68 on a PET substrate through replica molding. Finally, a TiO_2_ layer of gradient thickness (6.9 nm/mm) was deposited on a custom-made fixture through sputtering [20].

### 2.2. Design and Fabrication of Microfluidic Channels

In this study, a two-channel microfluidic chip was designed for the detection of biomolecules. One channel was used as a reference and the other was used for measurement. The microfluidic chip was fabricated using the polydimethylsiloxane (PDMS, Sylgard 184, Dow Corning) molding technique. In brief, a Poly (methylmethacrylate) (PMMA) mold (length, width, and height of 8, 5, and 3 mm, respectively) was fabricated using micromilling (EGX-400 engraving machine, Roland, USA). The gap between the two channels was 1 mm. Then, liquid PDMS with a base to curing agent ratio of 8:1 was poured on top of the PMMA mold. After degassing, the PDMS–PMMA structure was cured at 80 °C for 90 min in an oven. Finally, the PDMS was separated from the PMMA mold, and the inlet and outlet holes were created using a biopsy punch.

### 2.3. Integration of the GWT-GMR Sensor and Microfluidic Chip

The GWT-GMR sensor was then integrated with the microfluidic chip for detecting sucrose solution and biomolecules. We first attached the GWT-GMR sensor to a glass slide using NOA68 and UV exposure; the fabricated device is shown in Figure 1b. The PDMS microfluidic channel and GWT-GMR/glass were permanently bonded together using the oxygen plasma pretreatment technique [28]. In brief, both the PDMS and GWT-GMR/glass were exposed to oxygen plasma (PDC-32G, Harrick Plasma) under oxygen (99.6%) and pressure of 200 mTorr at 18 W for 180 s to generate silanol groups on both surfaces. The exposed surfaces were brought into contact to form permanent Si–O–Si bonds.

### 2.4. Assay Protocol

For detecting albumin or creatinine, the surface of the GWT-GMR sensor had to be functionalized to immobilize the antibodies of albumin and creatinine before the GWT-GMR/glass was bonded to the microfluidic chip. Therefore, the GWT-GMR/glass (Figure 1b) was first treated with oxygen plasma to enrich the hydroxyl groups on the TiO_2_ surface. Subsequently, epoxy silane (1% 3-glycidoxypropyl dimethoxysilane in toluene) was dispensed on top of the GWT-GMR surface for 60 min at room temperate, after which the surface was rinsed with acetone, ethanol, and deionized (DI) water; the surface was then finally blow dried with N_2_ gas. After silanization, the GWT-GMR/glass was bonded to the microfluidic chip as described previously.

A solution of 100 μg/mL of anti-albumin antibodies (CSB-PA00060E1Rb, Cusabio) or anti-creatinine antibodies (CRN12-A, Genemed Synthesis Inc., GSI, San Antonio, TX, USA) in phosphate-buffered saline (PBS) was injected into the measurement channel and incubated for 8 h. Through this approach, antibodies on the silanized GWT-GMR surface were immobilized. The antibody solution was then aspirated, and the measurement channel was rinsed using PBS with 0.05% Tween (PBS-T) thrice to remove unbounded antibodies. The measurement channel was then blocked in a solution of 1% casein (ab126587, Abcam) in PBS for 1 h at room temperature to minimize nonspecific binding during antigen incubation. The blocking solution was then aspirated, and the measurement channel was rinsed with PBS-T thrice. Finally, fresh PBS was injected into both channels, and the signals were measured as the baseline signals.

Standard curves were generated using various concentrations of purified albumin (CSB-NP000601h, Cusabio) and creatinine (02101423-CF, MP Bio) in the blocking buffer. Albumin solutions of six concentrations, from 500 μg/mL to 0.8 ng/mL in a five-fold dilution, and one blank (PBS only) solution were used. Creatinine solutions of concentrations ranging from 10 mg/mL to 1 μg/mL in a 10-fold dilution and a blank solution were also analyzed. The analyte solution (starting from the lowest concentration) was injected into the microfluidic channels and incubated for 20 min at room temperature. The sensor was then rinsed with PBS-T thrice. Lastly, fresh PBS was injected, and the signal was recorded with time. The same procedure was repeated for the other concentrations.

### 2.5. Detection Principle and Measurement Setup

As discussed in the previous section, the GWT-GMR sensor converts spectral information to spatial information on a CCD. To measure the transmitted intensity distribution, we employed a simple transmission setup, as shown in Figure 1a. Narrowband (full width at half, FWHM, of ~2.6 nm) light generated from a monochromator (DK242, Spectral Products) was coupled to a fiber. The light was transverse-magnetic polarized before it was incident on the microfluidic channel and the embedded GWT-GMR sensor. In this work, there was no lens between GWT-GMR and the CCD, and the distance between GWT-GMR and the CCD was approximately 3–4 mm. At normal incidence, the monochromatic light resonated and was reflected at a specific thickness (resonant thickness) of the GWT-GMR, whereas it was transmitted at other thicknesses, appearing as a dark band on the CCD, as shown in Figure 1d. The intensity distribution along a specific row (i.e., the white line in Figure 1d) was extracted, as shown in Figure 1e. The minimum intensity corresponding pixel (MICP) is related to the accurate resonant position, which is determined through curve fitting using the Lorentzian model obtained with OriginPro 2016. When the RI of the cover layer was changed (as well as *n_eff_*) owing to different concentrations of the bulk solution or surface-adsorbed biomolecules, the monochromatic light had to resonate at different thicknesses for Equation (1) to be satisfied. This phenomenon was observed directly from the shift in the dark band (Figure 1d) as well as the MICP (Figure 1e) on the CCD. The amount of shift is related to the change in sample concentration.

## 3. Results

### 3.1. Simulation Results for Bulk Solution

**Figure 2:**
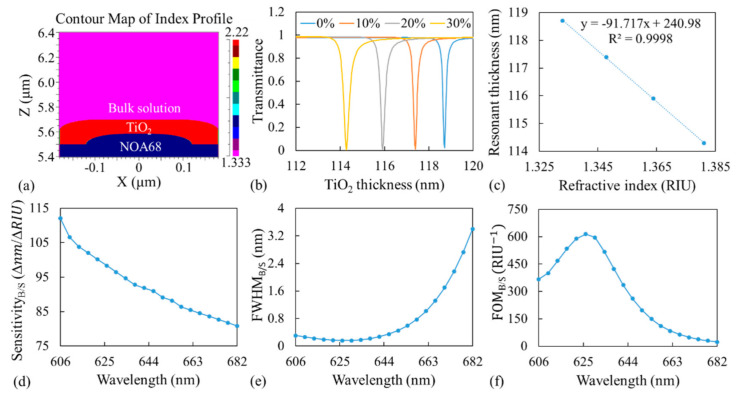
(**a**) Cross-section of one period of the simulated model obtained with DiffractMOD. (**b**) Transmittance as a function of TiO_2_ thickness for four different bulk concentrations at an incident wavelength of 626 nm. (**c**) Relationship between resonant thickness and bulk RI. (**d**) Sensitivity_B/S_, (**e**) FWHM_B/S_, and (**f**) FOM_B/S_ as a function of incident wavelength. Here, the subscript B/S indicates simulation results (S) obtained with the bulk solution (B).

We first used a simulation tool (DiffractMOD, RSoft Design Group) based on rigorous-coupled wave analysis to determine the optimal incident wavelength. Figure 2a shows an example of the cross-section of one cycle of a GMR structure in the simulation model (the *x*- and *y*-directions are not to scale) with rounded corners based on a scanning electron microscopy examination of the fabricated devices [20].

At a particular incident wavelength at a given bulk RI, by scanning the thickness in increments of 0.08 nm, we determined the transmittance as a function of TiO_2_ thickness, where the minimum transmittance corresponds to the TiO_2_ thickness at which resonance occurs. Figure 2b shows the transmittance as a function of TiO_2_ thickness for four sucrose solutions—0%, 10%, 20%, and 30%; the corresponding RIs were 1.333, 1.3478, 1.3638, and 1.3811, respectively [29], at an incident wavelength of 626 nm. The resonant thickness exhibited a linear relationship with the RI of a bulk solution as suggested by the coefficient of determination (R^2^) of 0.9998 of the linear fitted curve, as shown in Figure 2c.

Sensitivity was defined as the ratio of the lateral shift in resonant thickness to the RI variation. In other words, the sensitivity is the slope of the linear fitted line shown in Figure 2c; the sensitivity was determined to be −91.7 nm/RIU. The negative sign simply indicates that when the bulk RI increases, the light resonates at locations where the TiO_2_ layer is thinner to fulfill the resonant condition in Equation (1); hence, the absolute value is used to represent the sensitivity for a particular incident wavelength. The definition of sensitivity used in this study differs from that used in other relevant studies on spectral response, which is the shift in resonant wavelength with respect to the change in RI. The FWHM at 0% in Figure 2b was approximately 0.16 nm. The definition of FWHM in this study also differs from that in other studies. The FWHM in this study indicates the width of the resonant thickness, not the width of the resonant wavelength.

The same procedure was repeated for incident wavelengths of 606–682 nm in increments of 4 nm. The sensitivity and FWHM as functions of wavelength are shown in Figure 2d,e, respectively. The sensitivity and FWHM both exhibited opposite trends with respect to the wavelength. The sensitivity decreased with an increase in the incident wavelength, whereas the FWHM increased with the incident wavelength. The figure of merit (FOM), which is calculated by dividing the sensitivity (nm/RIU) with the FWHM, was adopted to evaluate the resonator-based optical biosensors from the spectral response [30]. We used the FOM to evaluate the GWT-GMR sensor for different incident wavelengths but with different definitions for sensitivity and FWHM. The results are shown in Figure 2f, which indicate an optimal incident wavelength of 626 nm.

To distinguish between the results in subsequent sections, we use two sets of subscripts. The first set of subscripts indicates whether the results pertain to the bulk solution (B) or surface-adsorbed layer (S). The second set of subscripts indicates whether the results are those of the simulation (S) or experiment (E). For example, in Figure 2d–f, the subscript B/S indicates simulation results obtained with the bulk solution.

### 3.2. Simulation Results for Surface Adsorption

The bulk sensitivity is a useful parameter when optical biosensors are employed for detecting large objects or when the RI of the test media fluctuates [31]. By contrast, the surface sensitivity is more useful for evaluating biosensors that require the immobilization of ligands, such as antibodies, to specifically capture target analytes [31,32]. Both sensitivities are not always correlated, and it is more practical to design optical biosensors based on surface sensitivity for detecting surface-bound biomolecules [31].

**Figure 3:**
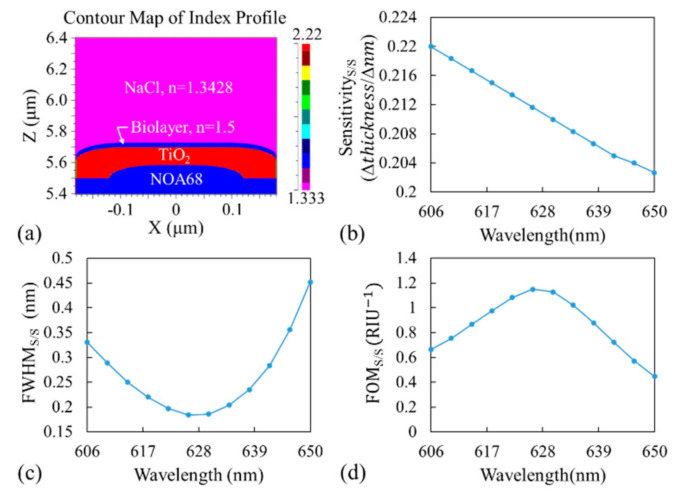
(**a**) Cross-section of one period of the simulated model with an additional biolayer on top of TiO_2_ to simulate biomolecule adsorption. (**b**) Sensitivity_S/S_, (**c**) FWHM_S/S_, and (**d**) FOM_S/S_ as a function of incident wavelength. Here, the subscript S/S indicates simulation results (S) obtained with the surface-adsorbed layer (S).

A 15 nm layer with an RI of 1.5 was added on top of the TiO_2_ layer surrounded by 0.9 M NaCl (n = 1.3428) to simulate the output response from surface-adsorbed biomolecules within the buffer solution, as shown in Figure 3a. Again, by scanning the TiO_2_ thickness, we obtained the transmittance as a function of TiO_2_ thickness and the corresponding resonant thickness (minimum transmittance) before and after the 15 nm layer was added. The surface sensitivity was then calculated as the ratio of the change in resonant thickness to the change in thickness (15 nm). The results are shown in Figure 3b, and they indicate that the surface sensitivity decreased with an increase in incident wavelength. The FWHM at the resonant thickness as a function of the incident wavelength is shown in Figure 3c. This figure indicates that an optimal incident wavelength is required to obtain the lowest FWHM to measure small shifts. The FOM_S/S_ for the surface deposition layer was defined as the ratio of surface sensitivity to the FWHM_S/S_. The results are shown in Figure 3d. Surprisingly, the optimal incident wavelength for both the bulk solution and the surface-adsorbed layer was 626 nm. In Figure 3b–d, the subscript S/S indicates simulation (S) results obtained with the surface-adsorbed (S) layer.

### 3.3. Bulk Solution of Sucrose Measurement

**Figure 4:**
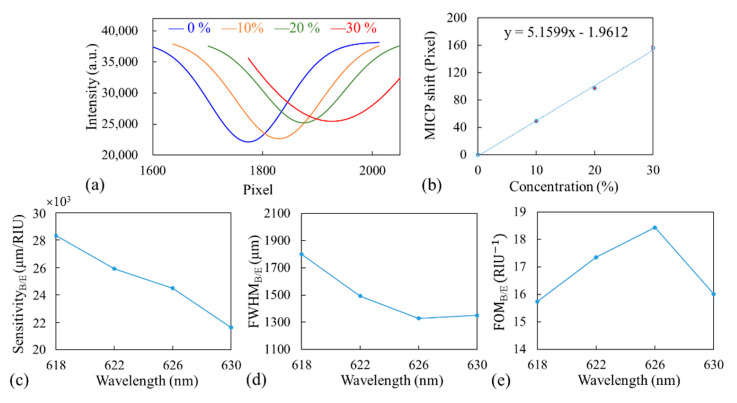
(**a**) Intensity distributions at different sucrose concentrations, and (**b**) shift in MICP as a function of concentration for the incident wavelength of 626 nm. (**c**) Sensitivity_B/E_, (**d**) FWHM_B/E_, and (**e**) FOM_B/E_ of the Scheme.

A GWT-GMR sensor (10 × 9 mm^2^) embedded in a microfluidic channel was used in bulk solution measurement. By measuring the thickness of TiO_2_ deposited on a piece of Si placed next to the GWT-GMR during gradient thickness deposition, the thickness of the GWT-GMR was approximately 63–132 nm. Four different concentrations of sucrose solution were used to verify the simulation results for bulk solution measurement. In the experiment, only DI water was initially injected into the microfluidic channel embedded with the GWT-GMR sensor, and the MICP was recorded as a reference. Sucrose up to 30% concentration was then added to the microfluidic channel in increments of 10%. After each measurement, the channel was rinsed with DI water. The process was repeated for another two runs. Figure 4a shows the fitted intensity distribution at different sucrose concentrations from one of the three runs. Figure 4b shows the shift in the MICP with respect to the MICP at 0% for all three runs at the incident wavelength of 626 nm. The sensitivity was approximated as the slope of the linear fitted line, that is, 5.16 pixels/% or 24,497 μm/RIU. The sensitivity refers to the actual lateral shift in MICP and cannot be compared with the sensitivity reported in the literature on spectral measurements. The measurement procedure was repeated for other incident wavelengths around the peak wavelength (626 nm) obtained in the simulations, and the results are shown in Figure 4b. Results similar to those obtained in the simulation (Figure 2d) were observed; the sensitivity decreased with an increase in the incident wavelength. The FWHM_B/E_ at 0% solution was selected to calculate the corresponding FOM_B/E_ for all four wavelengths; the results are shown in Figure 4c,d, and they indicate that the optimal incident wavelength was 626 nm.

### 3.4. Surface-Adsorbed Layer

Charged polyelectrolyte films were deposited on the GWT-GMR sensor to evaluate the response from the surface-adsorbed layer and to verify the simulation results. The polyelectrolytes used in this work were poly(ethyleneimine) (PEI, MW ~750,000), poly(sodium 4-styrenesulfonate) (PSS, MW ~70,000), and poly(allylamine hydrochloride) (PAH, MW ~17,000), which were all purchased from Sigma-Aldrich. The polyelectrolytes were all dissolved in 0.9 M NaCl at a concentration of 5 mg/mL. First, the PEI solution was pipetted into the GWT-GMR sensor and allowed to incubate for 5 min. After this, NaCl was used to rinse the sensor. This process was repeated for PSS and PAH in sequence. The thickness of each layer was approximately 5 nm [33]. The sensitivity_S/E_ was defined as the ratio of the shift in MICP before and after the deposition of PET/PSS/PAH to the total film thickness. The FWHM_S/E_ was determined from the transmittance vs. TiO_2_ thickness curve, similar to the manner in which it was determined in Section 3.1 before PET/PSS/PAH deposition. All measurements were performed with a bulk solution of NaCl.

**Figure 5:**
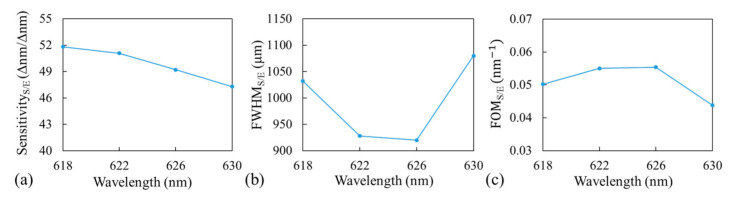
(**a**) Sensitivity_S/E_, (**b**) FWHM_S/E_, and (**c**) FOM_S/E_ from surface-adsorbed layer measurement at four incident wavelengths. Here, the subscript S/E indicates experimental results (E) obtained with the surface-adsorbed layer (S).

The sensitivity_S/E_, FWHM_S/E_, and FOM_S/E_ results for four different incident wavelengths (618, 622, 626, and 630 nm) around the peak wavelength obtained in the simulations are shown in Figure 5. Here, the subscript S/E indicates the experimental results obtained with the surface-adsorbed layer. Experimentally, the bulk solution and surface-adsorbed biolayer measurements conducted with our GWT-GMR sensor exhibited similar trends with the optimal wavelength of 626 nm; the results were in good agreement with the simulation results and hence were used for the detection of biomolecules.

### 3.5. Biomolecule Detection

Urine albumin and creatinine, which are commonly used biomarkers of chronic kidney disease (CKD) [34], were used to demonstrate the effectiveness of the proposed GWT-GMR sensor in practical biosensing applications. In this study, a two-channel microfluidic chip with the embedded GWT-GMR sensor (0.72 × 4 mm^2^) shown in Figure 1c was used to measure either albumin or creatinine concentration levels. One channel was used as a reference to compensate for any variation in the measurement system or experiment, and the other channel with immobilized antibodies was used to measure the binding between immobilized antibodies and the target analytes (albumin or creatinine). Measurements could be performed in both channels simultaneously, as shown in Figure 1d.

**Figure 6:**
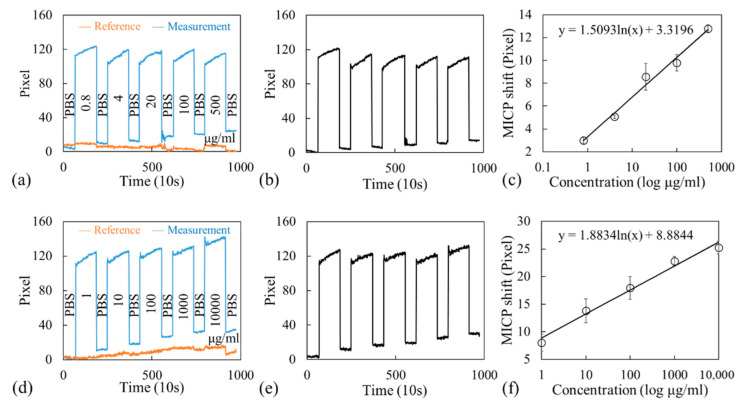
(**a**–**c**) Albumin and (**d**–**f**) creatinine detection results. (**a**,**d**) show the shift in MICP with respect to time for both the reference and measurement channels. (**b**,**e**) show the net shift in MICP with respect to time. (**c**,**f**) show the response curve.

Figure 6a shows the shift in MICPs with time for both the reference (orange curve) and measurement (blue curve) channels for albumin. Measurement started when PBS was immersed to stabilize the sensor, followed by the injection of 0.8 μg/mL albumin for 20 min. PBS-T was then used to rinse the unbound albumin, and fresh PBS was injected for the measurement, as indicated by the narrow slots in Figure 6a. During the measurement, the intensity distribution on the CCD was obtained every 10 s, and the MICPs were determined using the fitted curves exemplified in Figure 1e. The same procedure was repeated for other concentrations. The net shift in MICP with respect to time was calculated by subtracting the shift in MICP of the reference channel from that of the measurement channel; the results are shown in Figure 6b. The entire aforementioned procedure was performed on two other microfluidic chips embedded with GWT-GMR sensors, and the average shift in MICP for each concentration from three runs of experiments is shown in Figure 6c. The limit of detection (LOD) was calculated by multiplying the standard deviation of all measurements by three and then dividing the result by the sensitivity value, which was obtained from the slope (indicating sensitivity) of the graph shown in Figure 6c. The results indicated that the GWT-GMR sensor achieves an LOD of 2.92 μg/mL for albumin for the concentration range of 0.8–500 μg/mL investigated in this study.

In addition to testing the GWT-GMR sensor for detecting albumin, we tested the GWT-GMR sensor for detecting creatinine, which is another common biomarker associated with CKD. The procedure employed was similar to that employed for detecting albumin, except for the concentration range investigated. The results are shown in Figure 6d–6f, and they indicate that the GWT-GMR sensor achieved an LOD of 12.05 μg/mL for the concentration range of 1–10,000 μg/mL investigated in this study.

## 4. Discussion

In the simulation, the sensitivity (Figure 2d and Figure 3b) represents the ratio of the shift in MICP of the resonant thickness to the change in RI, and FWHM (Figure 2e and Figure 3c) represents the width in resonant thickness. Experimentally, the sensitivity (Figure 4c and Figure 5a) was calculated based on the actual MICP shift measured at the CCD, and FWHM (Figure 4d and Figure 5b) was also measured at the CCD, which depends on the TiO_2_ gradient. One may use a flatter TiO_2_ thickness gradient to increase the shift in MICP, which would in turn increase the sensitivity; however, this would increase the size of the sensor and broaden the FWHM_B/E_. Therefore, the sensitivity and FWHM values obtained from the simulation (Figure 2 and Figure 3) cannot be directly compared with those obtained from the experiment (Figure 4 and Figure 5). However, a large discrepancy was observed between the FOMs obtained through the simulation and experiment for both the bulk solution and surface-absorbed layer. We believe the main reason for this is the broad FWHM obtained in the experiment, which resulted in a low FOM. The FOM can be increased in the following manner. Firstly, an ultra-narrowband filter should be used to achieve a sub-nanometer linewidth, which would considerably reduce the FWHM measured at the CCD and increase the FOM. Secondly, other GMR designs with different fabrication processes should be adopted to achieve better resonance [24] because the corners of the device tend to be round when replica molding is used in the fabrication process, deteriorating the resonance and broadening the linewidth. Thirdly, the possible slightly diverging incident beam and the gap between the sensor and the CCD could also broaden the FWHM.

Compared to the chirped GMR based on graded duty cycle [19], current GWT-GMR exhibits a sensitivity of 24,497 μm/RIU, which is much higher than that of graded duty cycle GMR, at 3469 μm/RIU. This is mainly due to the flatter gradient achieved by the GWT-GMR. For the GWT-GMR, once the grating is replicated on the UV adhesive, the TiO_2_ thickness gradient can still be adjusted during sputtering deposition; hence, to achieve the desired sensitivity and detection range, which provides certain flexibility in terms of device fabrication.

An investigation of the urine albumin to creatinine ratio (UACR) is recommended for detecting microalbuminuria [35,36], which can be used to classify CKD and monitor its progress [37]. According to an extensive study conducted in the United States with 33,994 participants, the concentration of albumin in participants with a UACR of less than 300 μg/mg was between 0.7 and 50 μg/mL. These values were in the 5th–95th percentile range [35]. Another study with 577 general participants aged ≥40 years from Pakistan was between 2.1 and 8.5 μg/mL [38]. A Thai study showed that the creatinine concentration was approximately 390–2590 μg/mL in male participants and 280–2170 μg/mL in female participants [39]. Additionally, the creatinine concentration was 212–2763 μg/mL in the 5th–95th percentile of 33,994 U.S. participants with a UACR of less than 300 μg/mg [35].

We demonstrated that our proposed GWT-GMR sensor is capable of detecting albumin with an LOD of 2.92 μg/mL within a concentration range of 0.8–500 μg/mL, and that it is capable of detecting creatinine with an LOD of 12.05 μg/mL within a concentration range of 1–10,000 μg/mL. Although the detection of these recombinant proteins was demonstrated in buffer solutions, the GWT-GMR sensor exhibited considerable potential for detecting both albumin and creatinine in clinical settings [35,38,39]. We believe that by immobilizing different antibodies, the sensor can be used for numerous clinical screening or examination applications. Furthermore, the microfluidic chip can be further modified to include more channels for conducting multiple assays.

## 5. Conclusions

In this study, a GWT-GMR sensor was proposed as a novel type of LF biosensor. The sensor consisted of a replicated grating structure with a grating period of 360 nm and a TiO_2_ layer with a thickness gradient of 6.9 nm/mm. An incident wavelength of 626 nm was determined computationally and experimentally to achieve the best FOM in both the bulk solution and surface-adsorbed layer measurement.

By embedding the sensor in a two-channel microfluidic chip with measurement and reference channels, we minimized the potential disturbance from the measurement apparatus and interference from the environment. Although the FOM can be further optimized, we confirmed that through the detection of albumin and creatinine in a buffer solution, the current GWT-GMR sensor has considerable potential to achieve satisfactory results in clinical settings.

The GWT-GMR sensor represents a new paradigm for LF biosensing applications. Although the device performance can still be improved considerably, unlike uniform GMR sensors, the GWT-GMR sensor can convert spectral information directly into spatial information and record it on a CCD. This simplifies the design of the detection apparatus to a large extent; only a narrowband light source, such as an LED or laser diode, and a CCD or CMOS sensor are required. In addition, the replica molding on a plastic substrate and deposition of dielectric material ensures inexpensive sensor cost, which can be beneficial for disposable applications. We believe that the GWT-GMR sensor is suitable for use as a handheld device or integration with smartphones and could be beneficial for many point-of-care applications.

## Data Availability

Data available on reasonable request from the corresponding author.
